# Improvement of the Structure and Antioxidant Activity of Protein–Polyphenol Complexes in Barley Malts Using Roasting Methods

**DOI:** 10.3390/antiox14050538

**Published:** 2025-04-29

**Authors:** Guozhi Wu, Huiting Lin, Yongsheng Chen

**Affiliations:** Department of Food Science and Engineering, Jinan University, Guangzhou 510632, China; wuguozhi0930@163.com (G.W.); linhuiting23@163.com (H.L.)

**Keywords:** malt, protein–polyphenol complex, roasting, structure, antioxidant activity

## Abstract

Proteins and polyphenols are important components in barley malt. During the roasting process of barley malt, proteins and polyphenols interact and influence each other, ultimately altering the nutritional profile and functional properties of barley malt. In this research, polyphenol-free proteins and protein–polyphenol complexes were extracted from barley malt subjected to varying degrees of roasting. The antioxidant activity of protein–polyphenol complexes was assessed by ABTS, FRAP, and ORAC assays. The structural characteristics of the proteins were examined through UV, FL, CD, FTIR, and SEM. We found that roasting enhances the solubility of globulin, prolamin, and glutenin and facilitates the binding of these proteins with polyphenols. Conversely, the impact of roasting on albumin exhibits a trend opposite to that observed in the other three proteins. The antioxidant activity of protein–polyphenol complexes was significantly higher than that of polyphenol-free proteins. Additionally, the microenvironment of the amino acid residues of the four proteins exhibited increased polarity following the roasting process, and the structural conformation of albumin, globulin, and glutelin transitioned from an ordered to a disordered state. Our results indicate that roasting enhances the antioxidant activity of protein–polyphenol complexes by altering the secondary and tertiary structures of these proteins, thereby exposing more hydrophobic side-chain groups inside the proteins and offering more binding sites for polyphenols.

## 1. Introduction

Malt is the germinated grain of barley, which can be used not only as a raw material for brewing beer worldwide but also as a Chinese medicine following roasting [1]. The efficacy of malt is contingent upon its degree of roasting. Raw malt enhances lactation, roasted malt facilitates digestion, and burnt malt effectively invigorates the spleen [2]. Numerous studies have reported that malt has a range of bioactive properties, including anti-tumor activity, digestive enhancement, modulation of gut microbiota, hepatoprotection, glycemic regulation, anti-inflammatory effects, and antioxidant capabilities [3]. In recent years, malt has received increasing attention due to its notable health benefits, and its sales have been expanding year by year, with a variety of malt products emerging.

Malt contains abundant proteins, polysaccharides, and polyphenolic compounds, whose activity can be altered at high temperatures [4]. Therefore, malt can exhibit different health benefits after being roasted to different degrees [5]. For example, proteins in malt can undergo Maillard reactions during the roasting process, producing new colored substances such as Maillard reaction products (MRPs). MRPs have strong antioxidant activities and promote food digestion and absorption. Additionally, amino acids, including methionine, lysine, and tyrosine, within roasted malt proteins are susceptible to oxidation, leading to the antioxidant capacity of these proteins being impaired [6,7].

Polyphenolic compounds exhibit thermal instability, resulting in a progressive decline in their concentration across raw, roasted, and burnt malt. While water-soluble phenolic acids possess digestive-promoting properties, an excessive concentration of these acids may lead to gastrointestinal irritation. Raw malt is characterized by a high phenolic acid content, which can provoke gastrointestinal stimulation. In contrast, roasted malt mitigates this irritation to the gastrointestinal tract [8]. Roasted malt has been found to alleviate irritation in the gastrointestinal tract. Despite a reduction in the content of polyphenolic compounds following the roasting process, research indicates that within a specific temperature range, the antioxidant activity of these compounds is actually enhanced as the temperature rises.

Proteins and polyphenols in food can spontaneously interact through both non-covalent and covalent bonds [9]. The formation of complexes between proteins and polyphenols can inhibit various reactions that proteins undergo at elevated temperatures, such as the Maillard reaction and oxidative processes [10]. The interaction between proteins and polyphenols can enhance proteins’ interfacial activity, emulsification, and foaming properties, thereby improving the stability of proteins in food [11,12].

Compared with polyphenolic compounds, protein–polyphenol complexes demonstrate enhanced biological activities and stability, particularly anti-inflammatory and antioxidant functions, as well as the regulation of gut microbiota and the reduction of blood glucose levels [13]. Studies have indicated that elevated temperatures facilitate both non-covalent and covalent interactions between proteins and polyphenolic compounds, suggesting that protein–polyphenol complexes may exhibit increased antioxidant activity in roasted malt. However, there is little research addressing the variations in the activity of protein–polyphenol complexes in malt subjected to different degrees of roasting.

In the present study, we isolated polyphenol-free proteins and protein–polyphenol complexes from barley malt subjected to varying degrees of roasting. The impact of roasting degree on the component contents, antioxidant activity, and structure of polyphenol-free proteins and protein–polyphenol complexes were examined. This research aims to contribute a novel theoretical foundation for the advancement of malt-based food products.

## 2. Materials and Methods

### 2.1. Chemicals and Materials

Raw, roasted, and burnt malt (Table 1) were purchased from Huai Shun Tang, Bozhou, China. Coomassie brilliant blue R-250 was acquired from Shanghai Beyotime Biotechnology (Shanghai, China). 2,4,6-Tri (2-pyridyl)-1,3,5-triazine, Coomassie Brilliant Blue G-250, Trolox, and 2,2′-Azino-bis (3-ethylbenzothiazoline-6-sulfonic acid) were obtained from Shanghai Yuanye Bio-Technology Co., Ltd. (Shanghai, China). Tetramethylethylenediamine (TEMED), Tris-HCl (pH 8.8), Tris-HCl (pH 6.8), acrylamide, ferric chloride hexahydrate, sodium fluorescein, 2,2′-Azobis (2-methyl propionitrile) dihydrochloride, and ferrous sulfate were bought from Shnaghai Aladdin Biochemical Technology Co., Ltd. (Shanghai, China). BSA (purity ≥ 98%) was purchased from Beijing Megabion Biotechnology Co., Ltd. (Beijing, China). Ammonium persulfate, potassium persulfate, sodium lauryl sulfate (SDS), disodium hydrogen phosphate, sodium dihydrogen phosphate, sodium chloride, sodium hydroxide, potassium bromide, and hydrochloric acid were acquired from Tianjin Damao Chemical Reagent Co., Ltd. (Tianjin, China). All reagents were of analytical grade.

### 2.2. Preparation of Protein–Polyphenol Complexes and Polyphenol-Free Proteins

The extraction process was performed according to a published article, with appropriate minor modifications [14]. The Osborne method was employed to separate proteins into four polarity fractions: albumin, globulin, prolamin, and glutelin.

#### 2.2.1. Extraction of Protein–Polyphenol Complexes

The malt material was pulverized and suspended in deionized water (1:10, *w*/*v*). The mixture was ultrasonicated at room temperature for 15 min with an ultrasonic power of 300 W, magnetically stirred at room temperature (1 h), and centrifuged (3500 r/min, 20 min). The supernatant was filtered (500-mesh cloth) and subjected to sequential pH adjustments (4.3, 3.5, and 5.1) to precipitate albumin, globulin, and glutelin, respectively. Each precipitate was re-solubilized with deionized water, poured into a 300 Da molecular weight retention dialysis bag, and immersed in deionized water for 4 °C dialysis for 48 h. The final residue was extracted with 70% ethanol three times, centrifuged at room temperature (3500 r/min, 20 min), and the filtrate was adjusted to pH 4.7 to recover prolamin, followed by dialysis and lyophilization.

#### 2.2.2. Extraction of Polyphenol-Free Proteins

The malt material was pulverized and extracted with 80% aqueous acetone (1:10, *w*/*v*). The mixture was ultrasonicated at room temperature for 15 min with an ultrasonic power of 300 W, magnetically stirred (1 h), and centrifuged (10,000 r/min, 15 min). The supernatant was filtered (500-mesh cloth), rotary-evaporated, and adjusted to 50 mL with methanol to isolate free polyphenols. The residual protein was extracted using the same protocol described for protein–polyphenol complex extraction, yielding polyphenol-free protein fractions.

### 2.3. Determination of the Soluble Protein Content

The soluble protein content was determined using the Coomassie brilliant blue method [15]. Each protein sample was prepared at concentrations of 5 mg/mL. The sample solution (5 μL) was added to a 96-well plate, followed by the Coomassie brilliant blue staining solution (250 μL). The plate was incubated at 37 °C for 5 min, and absorbance was measured at 595 nm. The standard curve was plotted based on the concentration gradient and absorbance values of BSA.

### 2.4. Determination of Polyphenol Content

The total polyphenol content was quantified by the modified Folin–Ciocalteu method [16]. The diluted protein sample (10 μL) was added to a 96-well plate, along with a gallic acid standard solution (10 μL). Water (50 μL) and the Folin–Ciocalteu reagent (10 μL) were then added, mixed, and allowed to react for 3 min. A total of 30 μL of sodium carbonate solution was then mixed and allowed to react for 90 min. Absorbance was measured at 760 nm. PBS was used as the blank control. The standard curve was based on gallic acid concentrations and absorbance values. The total polyphenol content was expressed in equivalents of gallic acid (mg GAE/g).

### 2.5. ABTS+ Radical Scavenging Activity Assay

The ABTS+ radical scavenging activity assay was used to evaluate the radical scavenging activity of polyphenol-free proteins and protein–polyphenol complexes extracted from three types of malt based on a laboratory method [17]. Protein samples (20 μL) were mixed with the ABTS+ radical solution (380 μL) and allowed to react for 10 min at room temperature. Absorbance was measured at 734 nm using a microplate reader. The ABTS+ radical scavenging activity percentages of the samples are defined in Equation (1).

ABTS radical scavenging activity (%) = [1 − (A_sample_ − A_blank_)/A_control_] × 100 (1)

### 2.6. Ferric Ion-Reducing Antioxidant Power

The ferric ion-reducing antioxidant power (FRAP) method was used to assess the ferrous ion-reducing capacity of the four types of protein–polyphenol complexes and polyphenol-free proteins extracted from three types of malt [18]. A FRAP working solution was prepared by mixing sodium acetate buffer (0.3 M, pH 3.6), TPTZ solution (10 mM), and FeCl_3_ solution (20 mM) in a 10:1:1 ratio. The ferrous sulfate heptahydrate solution (1 mM) was prepared and diluted to concentrations of 0.01, 0.05, 0.1, 0.5, 1, and 1.5 mM for the standard curve. The FRAP working solution (180 μL) was added, and each protein sample or FeSO_4_ standard solution (5 μL) at various concentrations was added to a 96-well plate. The plate was incubated at 37 °C for 5 min, and absorbance was measured at 593 nm. The ferrous ion-reducing capacity was expressed relative to a 1 mM FeSO_4_ standard solution (1 mmol FeSO_4_ = 1 FRAP unit).

### 2.7. Oxygen Radical Absorbance Capacity

The oxygen radical absorbance capacity (ORAC) was determined [19]. First, 50 μL of samples, PBS (blank), and Trolox standard solution were added to a 96-well plate. The plate was incubated at 37 °C in a microplate reader for 10 min. A fluorescent working solution (100 μL) was then added to each well, and the plate was incubated for another 20 min. The AAPH working solution (90 μL) was added to all wells except the blank well, and fluorescence was determined immediately. Fluorescence signals were recorded over time, and the area under the curve (AUC) was calculated using Equation (2).

AUC = (0.5 × f₁/f₁ + f₂/f₁ + f₃/f₁ + … + f₃₄/f₁ + 0.5 × f₃₅/f₁) × CT(2)

where f_1_ is the initial fluorescence value, f_i_ is the fluorescence value at cycle i, and CT is the cycle time.

Net AUC values were obtained by subtracting the blank’s AUC from the AUC of the sample and Trolox. The final ORAC value was calculated using the linear regression of the net AUC versus the Trolox concentration and expressed as μmol Trolox equivalents (TE)/g of sample.

### 2.8. SDS-PAGE

SDS-PAGE was performed to determine the molecular weight of fractionated malt proteins, with modifications [20]. The protein solution was mixed with a 5× loading buffer at a 4:1 ratio, denatured and reduced by boiling at 100 °C for 5 min, and subsequently cooled to room temperature. After centrifugation, the supernatant was loaded onto a 12% separating gel and a 5% stacking gel. The marker (5 μL) and samples (20 μL) were loaded, and electrophoresis was conducted at 80 V until the sample entered the separating gel, then switched to 110 V until the bromophenol blue front reached 1 cm above the gel bottom. The gel was stained with Coomassie Brilliant Blue R-250 and destained until the protein bands were visualized before imaging.

### 2.9. Ultraviolet (UV) Spectroscopy

Protein samples were dissolved in phosphate buffer (PBS, pH 7.0) with proteins at a concentration of 0.125 mg/mL. UV spectra were recorded at room temperature in the range of 200–350 nm.

### 2.10. Intrinsic Fluorescence (FL) Spectroscopy

The intrinsic fluorescence spectra of protein samples were analyzed using a G9800A fluorescence spectrophotometer (Agilent, Shnghai, China). Proteins were dissolved in PBS (pH 7.0) to a final concentration of 0.125 mg/mL. Experiments were conducted at room temperature with excitation at 280 nm, and emissions were recorded in the range of 300–550 nm. Instrument settings included a voltage of 800 V and excitation and emission slits of 5 nm.

### 2.11. Fourier-Transform Infrared (FTIR) Spectroscopy

Samples were mixed with potassium bromide (KBr) at a ratio of 1:100. The mixture was milled into a fine powder and pressed into a thin pellet. FTIR spectra were recorded over the range of 4000–500 cm⁻^1^ with 32 scans. The data were analyzed using Omnic 8.2 software.

### 2.12. Scanning Electron Microscopy (SEM)

Samples of barley glutelin were mounted on conductive adhesive tape, sputter-coated with a 5 mm thick metal layer, and analyzed at an accelerating voltage of 20 kV under 2000× magnification to capture the microstructural features.

### 2.13. Circular Dichroism (CD) Spectroscopy

Samples were prepared at a concentration of 0.2 mg/mL. CD spectra were recorded in the range of 190–260 nm using a Chirascan CD spectrometer (Applied Photophysics Ltd., London, UK). Measurements were performed using a 1 mm path-length quartz cuvette at a scan speed of 100 nm/min and bandwidth of 0.1 nm. The average of three traces was calculated. Secondary structures were analyzed using CDNN software (version 2.1) within a wavelength range of 200–250 nm.

### 2.14. Amino Acid Content

The amino acid composition of proteins was analyzed using an L-8900 high-speed automatic amino acid analyzer (Hitachi, Tokyo, Japan). Samples were dissolved in 6 M HCl and subjected to acid hydrolysis at 110 ± 1 °C for 22 h in an electric heating air circulation oven. After hydrolysis, the filtrate was neutralized and dissolved in 0.02 M HCl for analysis. Results were reported as mmol/g of dry weight.

### 2.15. Statistical Analysis

All experiments were repeated three times, and the results are presented as mean values ± standard deviation. Intergroup differences are analyzed for significance using SPSS Statistics 27, with *p* < 0.05 considered significant. Origin 2023 software is used for data processing and analysis.

## 3. Results

### 3.1. Soluble Protein Content

**Figure 1:**
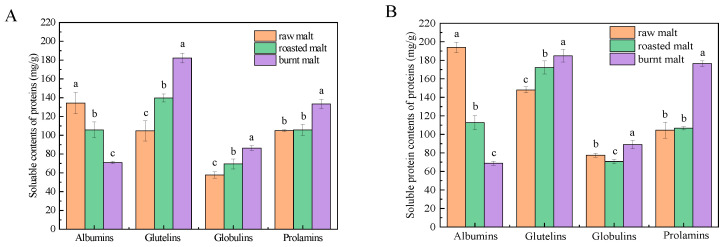

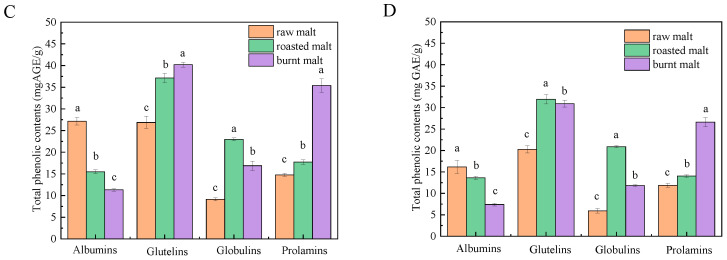
Soluble protein content of protein–polyphenol complexes (**A**) and polyphenol-free proteins (**B**) in raw, roasted, and burnt malt. Total polyphenol content of protein–polyphenol complexes (**C**) and polyphenol-free proteins (**D**) in raw, roasted, and burnt malt. The different letters marked in the figure indicated significant differences between the two groups of data (*p* < 0.05).

Soluble proteins were extracted from raw, roasted, and burnt malt, and their content was analyzed, as depicted in Figure 1A. As the degree of roasting increased, a significant decrease (*p* < 0.05) in the content of soluble albumins was observed. Specifically, the albumin content was highest in raw malt (134.31 ± 11.31 mg/g), followed by roasted malt (105.72 ± 8.36 mg/g), and lowest in burnt malt (71.07 ± 1.24 mg/g). Conversely, the content of soluble glutelins, globulins, and prolamins exhibited an increasing trend with higher roasting levels. The highest content of these proteins was found in burnt malt, measuring 182.17 ± 5.17 mg/g for glutelins, 86.39 ± 2.67 mg/g for globulins, and 133.29 ± 4.89 mg/g for prolamins. In contrast, the lowest concentrations were recorded in raw malt, with values of 104.63 ± 10.75 mg/g, 69.47 ± 4.89 mg/g, and 105.03 ± 0.9 mg/g, respectively.

We then investigated the levels of soluble proteins devoid of polyphenols. As depicted in Figure 1B, the elimination of polyphenols resulted in an increased content of soluble proteins in different malts. Notably, the overall pattern of soluble protein content, concerning the degree of roasting, remained consistent across both polyphenol-free proteins and protein–polyphenol complexes.

### 3.2. Total Polyphenol Content

Proteins were extracted from raw, roasted, and burnt malt, and the total polyphenol content within the protein–polyphenol complexes was quantified, as depicted in Figure 1C. A significant decrease in polyphenol content was observed in albumins with increasing roasting intensity (*p* < 0.05), with values recorded as follows: raw malt (27.13 ± 0.87 mg GAE/g) > roasted malt (15.50 ± 0.45 mg GAE/g) > burnt malt (11.33 ± 0.28 mg GAE/g). Conversely, the polyphenol content in glutelins and prolamins exhibited a significant increase with greater roasting degrees (*p* < 0.05). The highest polyphenol content in glutelins and prolamins was found in burnt malt (40.20 ± 0.58 mg GAE/g and 35.37 ± 1.55 mg GAE/g, respectively), while the lowest was observed in raw malt (26.87 ± 1.43 mg GAE/g and 11.87 ± 0.45 mg GAE/g, respectively). The polyphenol content in globulins was observed to be the highest in roasted malt (22.97 ± 0.35 mg GAE/g), followed by burnt malt (16.88 ± 1.07 mg GAE/g), and the lowest was observed in raw malt (9.13 ± 0.35 mg GAE/g).

The total polyphenol content was also measured in polyphenol-free proteins. The overall trend of polyphenol content in polyphenol-free proteins, as the degree of roasting increased, was largely consistent with that observed in protein–polyphenol complexes (Figure 1D). In raw malt, the polyphenol content was higher in albumin–polyphenol complexes and glutelin–polyphenol complexes, whereas it was relatively lower in globulin–polyphenol complexes and prolamin–polyphenol complexes.

### 3.3. ABTS+ Radical Scavenging Capacity

**Figure 2:**
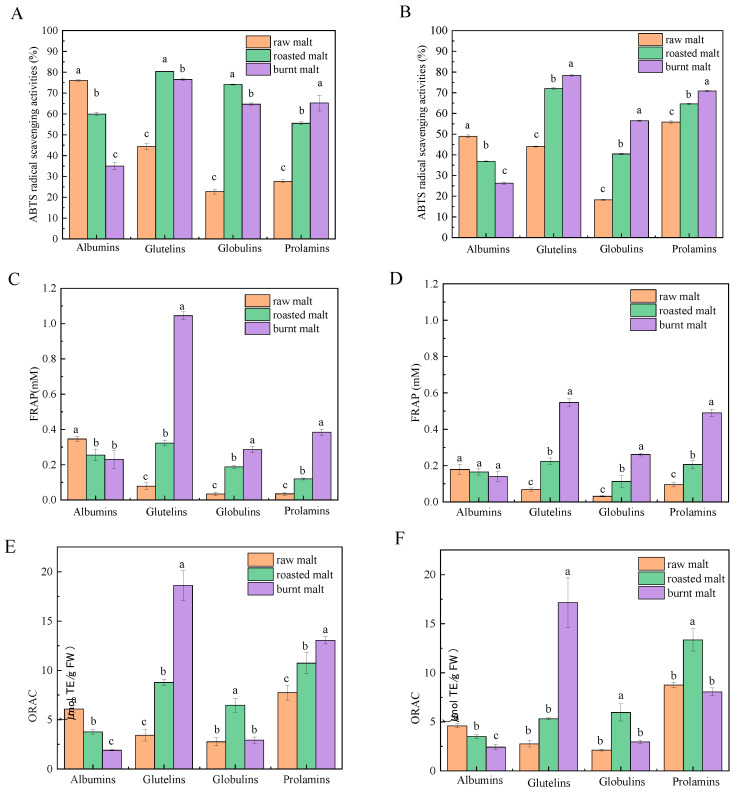
ABTS+ radical scavenging activities of protein–polyphenol complexes (**A**) and polyphenol-free proteins (**B**) in raw, roasted, and burnt malt. Ferric ion-reducing antioxidant power of protein–polyphenol complexes (**C**) and polyphenol-free proteins (**D**) in raw and roasted malt. Oxygen radical scavenging activities of protein–polyphenol complexes (**E**) and polyphenol-free proteins (**F**) in raw, roasted, and burnt malt. The different letters marked in the figure indicate a significant difference between the two groups of data (*p* < 0.05).

Proteins were extracted from raw, roasted, and burnt malt, and their ABTS+ radical scavenging capacity was measured, as depicted in Figure 2A. A significant decrease (*p* < 0.05) in the ABTS+ radical scavenging capacity of albumins was observed with increasing degrees of roasting, with values recorded as follows: raw malt (76.07 ± 0.33%) > roasted malt (59.96 ± 0.79%) > burnt malt (34.94 ± 1.78%). In contrast, the ABTS+ radical scavenging capacity of glutelins, globulins, and prolamins exhibited a significant increase with higher roasting degrees (*p* < 0.05). The highest polyphenol content in glutelins and globulins was observed in roasted malt, measuring 80.34 ± 0.19% and 74.12 ± 0.22%, respectively, followed by burnt malt at 76.49 ± 0.57% and 64.67 ± 0.57%, and the lowest values were seen in raw malt at 44.40 ± 0.14% and 22.74 ± 1.20%. The ABTS+ radical scavenging capacity of prolamins was the highest in burnt malt (65.21 ± 3.77%), followed by roasted malt (55.51 ± 0.63%), and the lowest was in raw malt (27.65 ± 0.72%).

The ABTS+ radical scavenging capacity of polyphenol-free proteins was also analyzed. The trend of the ABTS+ radical scavenging capacity of the protein–polyphenol complexes, as the degree of roasting increased, was largely consistent with that observed in polyphenol-free proteins. As depicted in Figure 2B, the elimination of polyphenols resulted in the decreased ABTS+ radical scavenging capacity of albumins and globulins in different malts.

### 3.4. Ferric Ion-Reducing Capacity

Proteins were extracted from raw, roasted, and burnt malt, and their ferric ion-reducing capacity was measured, as depicted in Figure 2C. Roasting significantly reduced the ferric ion-reducing capacity of albumins (*p* < 0.05). The ferric ion-reducing capacity of albumins was the highest in raw malt (0.35 ± 0.014 mM), followed by roasted malt (0.26 ± 0.034 mM), and the lowest in burnt malt (0.23 ± 0.051 mM). Conversely, the ferric ion-reducing capacity of glutelins, globulins, and prolamins increased significantly with increasing roasting degree (*p* < 0.05), while the highest was observed in burnt malt (1.05 ± 0.021 mM, 0.29 ± 0.019 mM, and 0.38 ± 0.017 mM, respectively), followed by roasted malt (0.078 ± 0.020 mM, 0.034 ± 0.0092 mM, and 0.035 ± 0.0082 mM, respectively), and the lowest in raw malt (0.32 ± 0.015 mM, 0.19 ± 0.0077 mM, and 0.12 ± 0.0057 mM, respectively).

The ferric ion-reducing capacity of polyphenol-free proteins was also detected. The trend of ferric ion-reducing capacity in the polyphenol-free glutelins, globulins, and prolamins, as the degree of roasting increased, was largely consistent with that observed in the protein–polyphenol complexes, as depicted in Figure 2D. Except for prolamins, the ferric ion-reducing capacity of polyphenol-free proteins was lower than in the protein–polyphenol complexes.

### 3.5. Oxygen Radical Scavenging Capacity

Proteins were extracted from raw, roasted, and burnt malt, and their oxygen radical scavenging capacity was measured, as depicted in Figure 2E. As the degree of roasting increased, a significant decrease (*p* < 0.05) in the oxygen radical scavenging capacity of albumins was observed, with values of 6.08 ± 0.95 μmol TE/g FW, 3.77 ± 0.25 μmol TE/g FW, and 1.90 ± 0.061 μmol TE/g FW. The oxygen radical scavenging capacity of glutelins and prolamins increased as the degree of roasting increased, measuring 18.62 ± 1.50 μmol TE/g FW and 10.72 ± 2.45 μmol TE/g FW in burnt malt, 8.78 ± 0.32 μmol TE/g FW and 10.74 ± 1.09 μmol TE/g FW in roasted malt, and 3.43 ± 0.59 μmol TE/g FW and 7.74 ± 0.76 μmol TE/g FW in raw malt. The oxygen radical scavenging capacity of globulins was the highest in roasted malt (6.47 ± 0.71 μmol TE/g FW), while no significant difference was observed between raw and burnt malt.

Additionally, the oxygen radical scavenging capacity of polyphenol-free proteins was also detected. The trend of oxygen radical scavenging capacity in the protein–polyphenol complexes, as the degree of roasting increased, was largely consistent with that observed in polyphenol-free proteins. In contrast, the oxygen radical scavenging capacity of the prolamins was the best in roasted malt. The oxygen radical scavenging capacity of polyphenol-free proteins was significantly lower than that of protein–polyphenol complexes (Figure 2F).

### 3.6. Relative Molecular Mass

**Figure 3:**
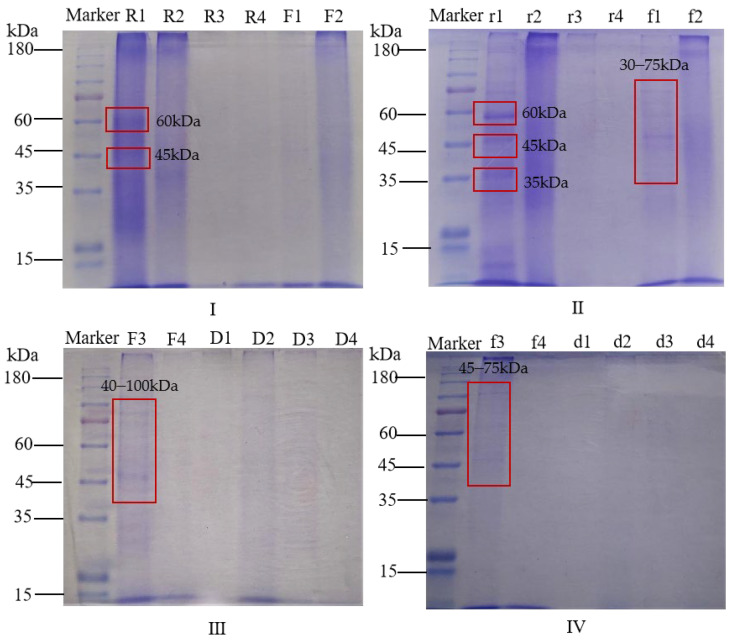
SDS-PAGE electrophoretic profiles of protein–polyphenol complexes and polyphenol-free proteins in raw, roasted, and burnt malt. (**I**) R1–R4 represent albumins, glutelins, globulins, and prolamins in raw malt; F1–F2 represent albumins and glutelins in roasted malt; (**II**) r1–r4 represent polyphenol-free albumins, glutelins, globulins, and prolamins in raw malt, respectively; f1–f2 represent polyphenol-free albumins and glutelins in roasted malt; (**III**) F3–F4 represent globulins and prolamins in roasted malt, and D1–D4 represent albumins, glutelins, globulins, and prolamins in burnt malt; (**IV**) f3–f4 represent polyphenol-free globulins and prolamins in roasted malt, d1–d4 represent polyphenol-free albumins, glutelins, globulins, and prolamins in burnt malt.

The subunit distribution of albumin–polyphenol complexes and polyphenol-free albumins in raw malt is depicted in Figure 3I (R1) and Figure 3II (r1), predominantly in the low molecular weight range at approximately 60, 45, and 35 kDa, with a notably pronounced band at 60 kDa. The electrophoretic mobility of the albumin–polyphenol complexes was observed to be lower than that of the polyphenol-free albumins, with all bands exhibiting a slight shift toward a higher molecular weight. The bands of globulins and prolamins were not distinct. The bands of glutelins (R2, r2) aggregated above 180 kDa. As the degree of roasting increased, the color of the protein bands also gradually became lighter (such as R1, F1, and D1).

### 3.7. Ultraviolet Spectroscopy

**Figure 4:**
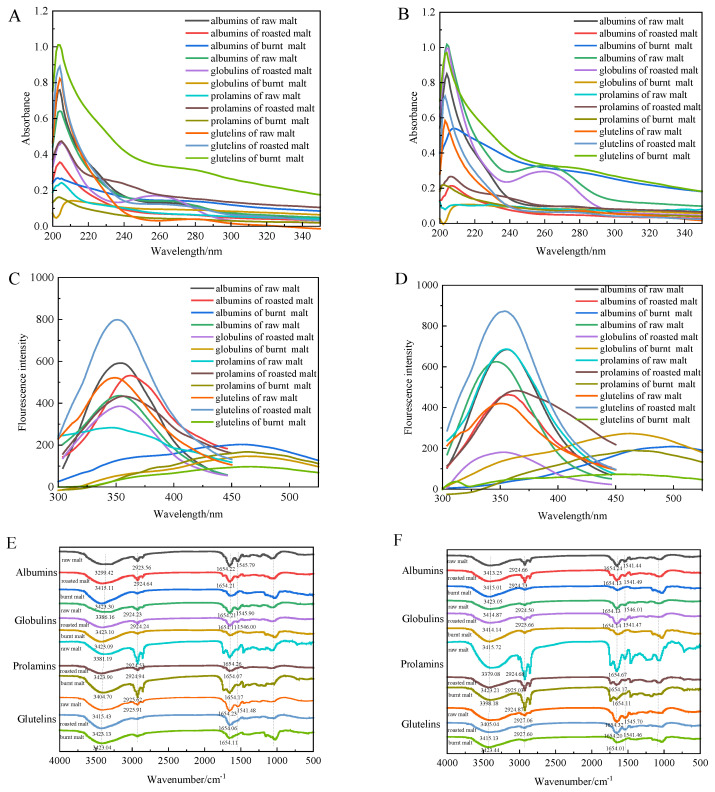
Ultraviolet spectroscopy of protein–polyphenol complexes (**A**) and polyphenol-free proteins (**B**) in raw, roasted, and burnt malt. Fluorescence spectroscopy of protein–polyphenol complexes (**C**) and polyphenol-free proteins (**D**) in raw, roasted, and burnt malt. Fourier-transform infrared spectroscopy of protein–polyphenol complexes (**E**) and polyphenol-free proteins (**F**) in raw, roasted, and burnt malt.

The ultraviolet spectroscopy of different proteins is shown in Figure 4A. Proteins contain aromatic amino acids such as phenylalanine, tyrosine, and tryptophan, thus having a maximum absorption wavelength of around 260–280 nm. In natural proteins, aromatic amino acid molecules that can produce fluorescence are mostly located inside the protein. The proteins had two ultraviolet absorption peaks at 200–220 nm and 260–280 nm, respectively, attributed to the absorption of the protein backbone structures C=O and Trp and other amino acid residues. Albumins in raw malt had a characteristic peak at 260 nm, and the intensity of this peak weakened as the degree of roasting increased. Conversely, as the degree of roasting increased, the peak intensity of albumins and globulins decreased, accompanied by a blue shift. The absorption peak of polyphenol-free globulins at 268 nm was significant and well-shaped, with the peak intensity of globulins in roasted malt being higher than that in raw malt. The absorption peak of prolamins at 280 nm was not significant. Glutelins had a characteristic peak at 280 nm, and the peak intensity significantly decreased as the degree of roasting increased, also incurring a blue shift.

The ultraviolet spectroscopy of polyphenol-free proteins is shown in Figure 4B. The ultraviolet absorption peaks of albumins in raw malt at 204 nm and around 260 nm both red-shifted, and the maximum absorption peak intensity changed. The ultraviolet absorption peak intensity of globulins at 260 nm in the polyphenol-free proteins increased, accompanied by a slight shift of the absorption peak.

### 3.8. Intrinsic Fluorescence Spectroscopy

Tryptophan and tyrosine provide intrinsic fluorescence for proteins, marking the strongest fluorescence emission peak of proteins in raw malt and roasted malt at around 350 nm, and proteins in burnt malt at around 460 nm (Figure 4C). As the degree of roasting increased, the fluorescence intensity of albumins, globulins, and polyphenol-free prolamins decreased. In contrast, the peak value of prolamins and glutelins first increased and then decreased as the degree of roasting increased.

However, the absorption peak of protein–polyphenol complexes still underwent a slight red shift as the degree of roasting increased. Compared with the protein–polyphenol complexes, the fluorescence intensity decreased in raw malt, but the trend of fluorescence intensity decreased after roasting disappeared (Figure 4D).

### 3.9. Fourier-Transform Infrared Spectroscopy

The Fourier-transform infrared spectroscopy of different malt proteins is shown in Figure 4E. The amide A band (near 3400 cm^−1^) represents the stretching vibrations of internal molecular hydrogen bonds and O–H and N–H; the amide B band (near 2925 cm^−1^) represents the vibration of C–H in CH_3_ and CH_2_ groups in the side chains of aliphatic amino acids; the amide I band (near 1650 cm^−1^) represents the C=O stretching vibration; the amide II band (near 1540 cm^−1^) represents the N–H deformation vibration and C–N and C–C bond stretching vibration; and the amide III band (near 1240 cm^−1^) represents the C–N stretching vibration and N–H bending vibration. As the degree of roasting increased, the amide A and amide B bands of different proteins underwent varying degrees of blue shifts, indicating that the stretching vibrations of O–H, N–H, and C–H bonds were weakened or masked. Except for prolamins, the intensity of the amide I and amide II bands of the other three proteins decreased, with the amide I band slightly red-shifted. Compared with protein–polyphenol complexes, the amide I band of polyphenol-free proteins showed no significant shift, but the amide II bands of albumins and globulins exhibited a significant red shift (Figure 4F).

### 3.10. Micromorphology

**Figure 5:**
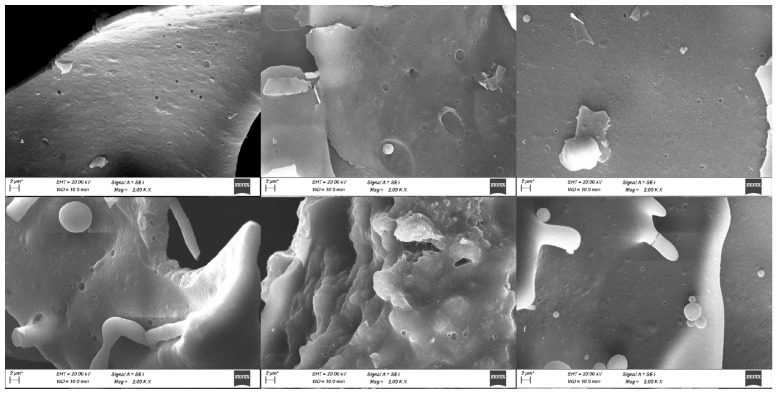
SEM of glutelin–polyphenol complexes in raw malt (**a**), roasted malt (**b**), and burnt malt (**c**). Polyphenol-free glutelins in raw malt (**d**), roasted malt (**e**), and burnt malt (**f**).

The surface morphology of gluten proteins affected by roasting was observed using scanning electron microscopy. The glutelins in raw malt had a flaky structure with many dense pores on the surface (Figure 5). The pores on the surface of glutelins in roasted malt became larger, and the pores on the surface of gluten proteins in burnt malt became more numerous and smaller. Compared with glutelins, the surface of polyphenol-free glutelins was rougher and more irregular.

### 3.11. Secondary Structure

The circular dichroism spectra of different malt proteins are shown in Figure S1. Albumins had two negative shoulder peaks at 208 nm and 222 nm, and polyphenol-free albumins had a positive peak at 203 nm. Globulins had two negative peaks at 205 nm and 222 nm. Prolamins and glutelins had a significant negative peak at 205 nm and an indistinct negative shoulder peak at 225 nm. With the increase in roasting degree, the negative peaks of malt proteins at 200–210 nm underwent shifts, which indicates the change in secondary structure.

The secondary structure of malt proteins is dominated by β-sheets and random coils (Table 2). As the degree of roasting increased, the content of α-helix in albumins decreased, and the content of β-sheets, β-turns, and random coils increased. The content of α-helix in globulins decreased, and the content of β-sheets, β-turns, and random coils showed a trend of first decreasing and then increasing, while the content of random coils in polyphenol-free globulins increased successively. The secondary structure of prolamins changed little after roasting. As the degree of roasting increased, the α-helix structure of glutelins decreased, β-sheets decreased, and random coils increased, while the α-helix structure of polyphenol-free glutelins decreased while β-sheets and random coils increased.

Compared with the protein–polyphenol complexes, the α-helix content of polyphenol-free albumins and globulins of raw malt increased, and the content of β-sheets, β-turns, and random coils decreased; the α-helix content of glutelins decreased, and the content of β-sheets, β-turns, and random coils increased (Table 2).

### 3.12. Amino Acid Content

The amino acid composition of malt was detected using an amino acid automatic analyzer, and the results are shown in Table 3. Seventeen amino acids (Asp, Thr, Ser, Glu, Gly, Ala, Cys, Val, Met, Ile, Leu, Tyr, Phe, Lys, His, Arg, and Pro) were detected in malt proteins. Cys was not detected in some proteins. There were significant differences in the amino acid content of raw, roasted, and burnt malt, and the total amino acid content generally showed a downward trend with the increase in roasting degree (*p* < 0.05). The total amino acid content was the highest in roasted malt and the lowest in raw malt. Albumins and globulins had the highest content of aspartic acid, glutamic acid, alanine, and leucine. Prolamins had the highest content of glutamic acid, proline, and phenylalanine. The tyrosine and valine content was the highest in burnt malt, while the cysteine and proline content was the highest in roasted malt. Glutelins had the highest content of glutamic acid, leucine, and proline, and there was no significant difference in amino acid content between raw malt and roasted malt, although the content of glutamic acid and leucine in roasted malt was higher than that in raw malt. The total amino acid content of polyphenol-free proteins was higher than that of protein–polyphenol complexes.

## 4. Discussion

Malt, as an important cereal raw material, plays an irreplaceable role in the food and brewing industries. It is rich in proteins and polyphenols, and thermal processing can significantly influence the characteristics of these components, altering their physicochemical properties and functionalities [21,22]. This study employs multispectral technology, combined with antioxidant activity assays and amino acid composition analysis, to demonstrate that roasting not only modifies the structure of proteins but also alters the interaction between polyphenols and proteins. Moreover, roasting enhances the solubility and antioxidant capacity of globulins, prolamins, and glutelins. These findings deepen our understanding of protein–polyphenol interactions during malt processing and provide valuable theoretical and practical guidance for optimizing malt processing techniques and improving the functional properties of malt-based products.

Roasting significantly increased the solubility of globulins, prolamins, and glutelins, with the most pronounced effect observed on glutelins. However, the solubility of albumins decreased during roasting. Previous studies have reported that baking treatment enhances the solubility of pea protein isolate (the main protein was globulin) by approximately 12% [23]. This is consistent with our findings of increased globulin solubility in malt. Conversely, a significant reduction in globulin content after roasting was observed, attributed to its thermal degradation into smaller peptides [24]. SDS-PAGE results revealed clearer subunit bands in globulins after roasting, suggesting that thermal treatment promoted the formation of soluble protein aggregates, thereby improving globulin’s migration properties. Similarly, roasting significantly altered the migration characteristics of globulin subunits in beans [23]. We hypothesize that the structural rearrangement of globulins during roasting may facilitate the formation of soluble aggregates, thereby enhancing solubility. This aggregate formation occurs due to the disruption of intermolecular hydrogen bonds during heating, which are not removed by centrifugation [25]. Consistent with our findings, increased prolamin and glutelin content and decreased albumin content in roasted chickpeas were observed [26]. A reduction in soluble albumin content in roasted cashews was also noted [27]. The decline in the solubility of albumin is likely due to heat-induced thermal denaturation and aggregation [28]. Albumins are water-soluble, low-molecular-weight proteins with loose structures prone to aggregation. Under high-temperature conditions, they undergo denaturation, including the sequential dissociation of subunits and the reaggregation of partially unfolded molecules, ultimately forming insoluble complexes [24,26]. Additionally, the solubility of four types of proteins in complexes was found to be higher compared to corresponding polyphenol-free proteins, similar to the results reported by others [29,30].

After roasting, the content of polyphenols in globulin, prolamin, and glutelin extracts significantly increased, whereas the polyphenol content in albumin extracts decreased. This phenomenon can be attributed to the thermal denaturation of proteins and their interaction with polyphenols [31]. Firstly, globulins, glutelins, and prolamins exhibit high hydrophobicity and structural stability, making them more prone to forming stable associations with polyphenols under high temperatures. This interaction may be strengthened through non-covalent bond mechanisms such as hydrophobic interactions or hydrogen bonding, thereby reducing phenolic oxidation or decomposition during heating and ultimately increasing polyphenol retention in protein extracts [32,33]. These findings are consistent with a study reporting that roasting significantly increased total phenolic content in chickpea protein isolate [34]. Additionally, the increased phenolic content correlates with the elevated formation of Maillard reaction products during roasting, which share structural similarities with phenolic compounds and can be detected as phenolic compounds [35].

In contrast, the observed decline in polyphenol content in albumin extracts is likely due to the thermal denaturation of albumins, as evidenced by the fading of their electrophoretic bands, indicating significant protein aggregation. High temperatures can induce protein aggregation via disulfide bond rearrangements, increased intermolecular β-sheet formation, and enhanced intermolecular interactions [36]. This result aligns with studies on peanut albumins [37] and oat albumins [38], both of which observed similar effects under thermal processing conditions. We speculate that the thermal instability of albumins disrupts their inherent ability to bind polyphenols, thereby releasing previously bound polyphenols. In raw malt, albumin extracts exhibit a higher polyphenol content, likely because albumins readily associate with polyphenols at neutral pH levels [39]. The bands of the albumin–polyphenol complex exhibit a slight shift in molecular weight, indicating that their binding increased their molecular weight, further confirming the stable association of albumins with polyphenols in raw malt [40].

The antioxidant activity of globulins, prolamins, and glutelins significantly increased after roasting, while the antioxidant activity of albumins decreased. Roasting enhanced the DPPH radical and hydrogen peroxide scavenging activity of chickpea protein isolates [34]. Similarly, thermal processing significantly improved ferric ion-reducing power in legume proteins [36]. These findings align closely with our results, indicating a consistent increase in antioxidant activity for globulins, prolamins, and glutelins. The enhanced antioxidant activity can be attributed to the strengthened synergies between proteins and polyphenols. Both UV and fluorescence spectroscopy results demonstrated that roasting significantly influenced the tertiary structure of globulins and glutelins in malt. High-temperature treatment might induce the structural rearrangement of proteins, exposing hydrophobic amino acids and sulfur-containing residues (Trp, Phe, Tyr, and Cys), thereby polarizing their microenvironments. This unfolded structure provides more effective binding sites for polyphenols, as previously reported [14,27]. Similar findings were reported in yellow pea proteins, which showed significant alterations in their tertiary structures upon roasting [23]. The elevated temperature accelerates domain reorganization, enhances the exposure of hydrophobic amino acids, and strengthens intermolecular interactions, all of which contribute positively to the antioxidant activity of proteins [32,41].

However, the secondary structure composition of prolamins remained nearly unchanged during roasting, which might be due to their high thermal stability and hydrophobicity. Compared to albumins and globulins, prolamins maintain better preservation of their secondary structural integrity under roasting. This suggests that physical characteristics, such as hydrophobicity, play a critical role in protein behavior during thermal processing. Numerous studies have demonstrated that antioxidant activity is closely associated with changes in the secondary structures of proteins [32,36]. The disordered structure of proteins facilitates the exposure of active regions, and a transition from ordered structures to random coils often correlates with functional activity changes [42].

Glutelins exhibited particularly outstanding antioxidant activity after roasting. SEM results revealed minimal changes in their microstructure but an increase in porosity after roasting, which may enhance the affinity and distribution of polyphenols, thereby further boosting antioxidant activity [43]. The secondary structure transition from ordered α-helix and β-sheets to disordered β-turns and random coils suggests significant denaturation of glutelins under roasting, accompanied by structural unfolding. Heat treatment leads to a greater disordered structure in protein secondary structures. Similarly, thermal processing induced the transformation of α-helix into β-turns of proteins in barley, which was consistent with our findings [44].

In contrast to the other three proteins, the antioxidant activity of albumins and their complexes significantly decreased after roasting. Fluorescence spectra revealed a significant fluorescence quenching effect of polyphenols in albumin extracts, indicating that polyphenols may influence protein structure through mechanisms such as electrostatic interactions, hydrophobic interactions, or hydrogen bonding. Compared to raw malt, the disappearance of fluorescence quenching in roasted malt suggests that high-temperature treatment caused irreversible structural changes in albumins, weakening the interaction between polyphenols and albumins and facilitating the release or degradation of bound polyphenols [45]. This change not only reduced the polyphenol content in albumin extracts but also diminished their antioxidant capacity. Similar results in purple rice proteins were observed, showing that albumins exhibit higher antioxidant activity in raw malt [46]. This may be due to the enhanced ability of albumins in raw malt to bind with polyphenols, thereby displaying stronger antioxidant properties.

It is worth noting that the antioxidant activity of protein–polyphenol complexes significantly exceeded that of polyphenol-free proteins. Polyphenols considerably altered the three-dimensional structure of proteins, inducing a more polarized microenvironment for amino acids in albumins, globulins, glutelins, and prolamins [41]. The interaction between polyphenols and proteins protected polyphenols from oxidation, thereby enhancing the antioxidant performance of the complexes. Thermal treatment did not disrupt the interaction between cyanidin-3-O-glucoside (C3G) and β-lactoglobulin (β-Lg) but instead significantly improved the antioxidant activity of β-Lg through synergistic effects [47].

## 5. Conclusions

This study investigated the structure and antioxidant activity of polyphenol-free proteins and protein–polyphenol complexes in malt at different roasting degrees. Roasting significantly affected the solubility of protein and polyphenol content, with distinct protein-type specificity observed. The antioxidant activity of albumin decreased with increasing roasting degrees, possibly due to the disruption of its interaction with polyphenols during roasting. Roasting altered the secondary and tertiary structures of both polyphenol-free proteins and protein–polyphenol complexes, leading to protein unfolding and the exposure of hydrophobic side chain groups (Tyr, Trp, and Phe), which increased the antioxidant activity of globulin, prolamin, and glutelin. Notably, the protein–polyphenol complexes exhibited significantly higher antioxidant activity compared to polyphenol-free proteins. Furthermore, the antioxidant activity of protein–polyphenol complexes improved after roasting, indicating potential applications in advancing functional foods and utilizing roasted malt for diverse effects. These findings provide significant insights into the structural and functional changes in malt proteins and polyphenols under roasting.

Natural products are abundant in barley malt, where the interactions and influences among these components are intricate. Additionally, the effects of roasting on the diverse components are highly variable. In our current research, we have preliminarily demonstrated the interaction between proteins and polyphenols, laying a foundation for further exploration of their interaction and functional mechanisms. Notably, in vitro antioxidant assays on natural products are insufficient to fully explain the antioxidant effects of protein–polyphenol complexes within the human body. Therefore, future research could incorporate in vivo experiments to clarify the absorption, distribution, and metabolism of these complexes. Such investigations will contribute to maximizing the nutritional value of barley malt and facilitate the development of food products with enhanced functionality and nutritional profiles.

## Figures and Tables

**Table 1:** Descriptions of different barley malts.

Picture	Malt Name	Chinese Name	Description	Preparation Method
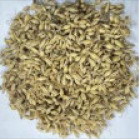	Raw malt	Sheng maiya	Light yellow in color with a hard texture, light smell, and sweet taste.	Ripe barley grains are soaked until germination and dried when the buds reach 5 mm.
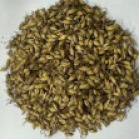	Roasted malt	Chao maiya	Brown-yellow in color with small char spots, slightly bitter in aroma and taste.	The raw malt is stir-fried to produce a brown-yellow color.
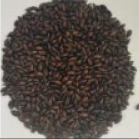	Burnt malt	Jiao maiya	Dark brown color, with a dark aroma and slightly bitter taste.	The raw malt is fried until dark brown.

**Table 2:** Secondary structure content of protein–polyphenol complexes and polyphenol-free proteins in malt.

Samples		α-Helix (%)	β-Turn (%)	Random Coil (%)	β-Sheet (%)
Albumin-polyphenol complexes	raw malt	12.2	18.0	42.3	29.9
	roasted malt	11.7	18.3	42.7	30.8
	burnt malt	10.4	18.7	44.8	33.4
Globulin-polyphenol complexes	raw malt	10.8	18.6	44.2	32.6
	roasted malt	11.2	18.4	43.8	31.9
	burnt malt	10.4	18.7	44.9	33.4
Prolamin-polyphenol complexes	raw malt	10.5	18.7	44.9	33.4
	roasted malt	10.6	18.6	44.5	33.1
	burnt malt	10.5	18.7	44.8	33.3
Glutelin-polyphenol complexes	raw malt	11.8	18.2	42.0	36.4
	roasted malt	11.2	18.4	43.4	31.7
	burnt malt	10.5	18.7	44.6	33.1
Polyphenol-free albumins	raw malt	14.8	17.2	37.3	30.4
	roasted malt	11.6	18.2	43.1	30.9
	burnt malt	10.4	18.7	45.0	33.6
Polyphenol-free globulins	raw malt	12.7	17.8	41.8	29.0
	roasted malt	12.0	17.4	43.7	28.4
	burnt malt	10.4	18.7	45.1	33.6
Polyphenol-free prolamins	raw malt	10.5	18.7	44.9	33.4
	roasted malt	10.3	18.7	45.2	33.5
	burnt malt	10.5	18.7	44.8	33.4
Polyphenol-free glutelins	raw malt	11.2	18.4	43.4	31.7
	roasted malt	11.4	18.3	42.9	31.3
	burnt malt	10.6	18.7	44.5	33.1

**Table 3:** Content of amino acids (mg/g) of protein–polyphenol complexes and polyphenol-free proteins in malt.

	Raw Malt	Roasted Malt	Burnt Malt	Raw Malt	Roasted Malt	Burnt Malt	Raw Malt	Roasted Malt	Burnt Malt	Raw Malt	Roasted Malt	Burnt Malt	
	**Albumin**-polyphenol complexes			**Globulin**-polyphenol complexes			**Prolamin**-polyphenol complexes			**Glutelin**-polyphenol complexes			
Asp	43.7 ± 2.4 a	18.2 ± 0.6 b	1.3 ± 0.0 c	43.9 ± 3.9 a	19.4 ± 0.2 b	3.2 ± 0.0 c	3.2 ± 0.4 b	3.7 ± 0.1 b	6.3 ± 0.1 a	36.7 ± 2.7 a	29.7 ± 1.7 b		5.0 ± 0.2 c
Thr	20.6 ± 0.7 a	9.3 ± 0.2 b	0.4 ± 0.0 c	20.4 ± 1.6 a	9.1 ± 0.1 b	1.2 ± 0.0 c	2.9 ± 0.3 b	4.0 ± 0.1 a	1.0 ± 0.0 c	20.4 ± 1.4 a	18.4 ± 0.9 b		0.8 ± 0.0 c
Ser	18.8 ± 0.8 a	9.9 ± 0.3 b	0.4 ± 0.0 c	20.7 ± 1.8 a	9.0 ± 0.2 b	1.2 ± 0.0 c	4.6 ± 0.6 b	9.1 ± 0.3 a	0.3 ± 0.0 c	24.1 ± 1.7 a	22.5 ± 1.3 a		0.4 ± 0.0 b
Glu	60.5 ± 2.0 a	45.1 ± 1.3 b	6.3 ± 0.2 c	66.3 ± 4.9 a	34.6 ± 0.4 b	9.9 ± 0.2 c	30.4 ± 3.3 c	129.1 ± 2.6 a	49.9 ± 1.8 b	76.5 ± 1.8 a	103.4 ± 1.2 b		33.3 ± 1.7 c
Gly	19.2 ± 0.6 a	10.9 ± 0.2 b	0.80 ± 0.0 c	20.7 ± 0.9 a	10.7 ± 0.4 b	2.1 ± 0.1 c	3.1 ± 0.2 b	2.5 ± 0.1 c	6.1 ± 0.1 a	19.3 ± 1.1 a	19.1 ± 1.2 a		3.9 ± 0.1 b
Ala	36.7 ± 8.3 a	14.0 ± 0.3 b	1.2 ± 0.1 c	35.6 ± 3.0 a	15.3 ± 0.6 b	3.2 ± 0.1 c	3.9 ± 0.5 b	3.8 ± 0.2 b	9.2 ± 1.0 a	31.2 ± 1.2 a	25.8 ± 3.4 a		5.9 ± 0.3 b
Cys	N.d.	1.4 ± 0.1 a	0.1 ± 0.0 b	N.d.	N.d.	0.1 ± 0.0 a	N.d.	0.2 ± 0.0 b	0.5 ± 0.0 a	N.d.	N.d.		0.3 ± 0.0 a
Val	25.6 ± 0.9 a	11.8 ± 0.4 b	1.3 ± 0.0 c	28.1 ± 2.1 a	11.8 ± 0.2 b	2.7 ± 0.0 c	5.8 ± 0.6 c	7.2 ± 0.1 b	13.2 ± 0.4 a	29.5 ± 1.8 a	27.1 ± 1.5 a		7.5 ± 0.3 b
Met	6.8 ± 0.7 a	2.0 ± 0.0 b	0.02 ± 0.0 c	4.3 ± 0.4 a	2.9 ± 0.0 b	0.04 ± 0.0 c	N.d.	N.d.	N.d.	1.3 ± 0.0 b	1.9 ± 0.1 a		0.1 ± 0.0 c
Ile	20.1 ± 0.5 a	9.0 ± 0.3 b	0.8 ± 0.0 c	20.8 ± 1.2 a	8.8 ± 0.1 b	1.8 ± 0.1 c	5.0 ± 0.6 c	12.0 ± 0.3 a	10.3 ± 0.3 b	22.8 ± 1.6 a	20.5 ± 1.2 a		5.2 ± 0.2 b
Leu	44.5 ± 0.8 a	17.7 ± 0.2 b	1.6 ± 0.1 c	39.2 ± 0.7 a	16.8 ± 0.5 b	3.5 ± 0.1 c	9.3 ± 0.9 b	19.7 ± 0.9 a	20.7 ± 0.6 a	47.8 ± 0.4 a	41.9 ± 0.9 b		11.5 ± 0.5 c
Tyr	14.4 ± 0.5 a	7.3 ± 0.3 b	0.5 ± 0.0 c	15.1 ± 1.2 a	6.7 ± 0.0 b	1.1 ± 0.0 c	2.9 ± 0.3 c	12.0 ± 0.3 a	7.9 ± 0.2 b	18.1 ± 1.2 a	19.5 ± 1.3 a		4.1 ± 0.2 b
Phe	21.1 ± 0.8 a	13.0 ± 0.4 b	1.1 ± 0.0 c	24.0 ± 1.8 a	10.7 ± 0.0 b	2.2 ± 0.0 c	6.6 ± 0.7 c	40.7 ± 0.8 a	16.5 ± 0.4 b	31.7 ± 1.9 a	29.0 ± 1.5 a		8.7 ± 0.3 b
Lys	29.8 ± 1.2 a	10.0 ± 0.3 b	0.7 ± 0.1 c	26.5 ± 0.6 a	10.7 ± 0.5 b	1.8 ± 0.0 c	2.7 ± 0.2 a	1.4 ± 0.2 b	2.3 ± 0.0 a	17.3 ± 0.3 a	13.1 ± 0.0 b		1.7 ± 0.2 c
His	9.8 ± 0.6 a	5.1 ± 0.2 b	0.3 ± 0.0 c	10.4 ± 0.5 a	4.8 ± 0.2 b	0.8 ± 0.0 c	1.5 ± 0.0 c	4.2 ± 0.1 a	2.6 ± 0.2 b	11.8 ± 0.6 a	11.6 ± 0.8 a		1.6 ± 0.0 b
Arg	23.9 ± 1.1 a	12.9 ± 0.0 b	0.4 ± 0.0 c	30.8 ± 2.7 a	14.4 ± 0.6 b	1.5 ± 0.1 c	5.0 ± 0.5 b	8.6 ± 2.5 a	1.0 ± 0.1 c	25.8 ± 1.2 a	24.6 ± 1.5 a		0.3 ± 0.0 b
Pro	11.8 ± 0.3 b	13.8 ± 0.3 a	2.1 ± 0.1 c	12.7 ± 0.9 a	8.7 ± 0.1 b	3.0 ± 0.1 c	10.4 ± 0.9 c	80.8 ± 0.6 a	19.5 ± 0.6 b	39.8 ± 2.0 a	44.2 ± 2.1 a		11.5 ± 0.5 b
Total	407.5 ± 2.0 a	211.4 ± 5.3 b	19.4 ± 0.4 c	419.5 ± 20.9 a	194.3 ± 2.6 b	39.4 ± 0.7 c	97.2 ± 9.4 c	339.2 ± 9.1 a	167.1 ± 5.1 b	452.3 ± 20.6 a	464.3 ± 20.8 a		101.7 ± 4.6 b
	Polyphenol-free **albumins**			Polyphenol-free **globulins**			Polyphenol-free **prolamins**			Polyphenol-free **glutelins**			
Asp	20.2 ± 1.1 a	18.9 ± 0.6 a	1.2 ± 0.0 b	51.4 ± 3.3 a	24.2 ± 0.5 b	1.5 ± 0.2 c	2.6 ± 0.1 b	1.9 ± 0.0 c	4.5 ± 0.2 a	46.3 ± 1.3 a	34.1 ± 0.8 b		6.2 ± 0.1 c
Thr	9.7 ± 0.5 a	10.2 ± 0.3 a	0.3 ± 0.0 b	25.6 ± 1.5 a	14.7 ± 0.4 b	0.5 ± 0.1 c	2.4 ± 0.1 a	2.0 ± 0.0 b	0.7 ± 0.0 c	26.5 ± 0.3 a	19.6 ± 0.4 b		1.0 ± 0.0 c
Ser	10.8 ± 0.6 a	11.5 ± 0.4 a	0.3 ± 0.0 b	21.9 ± 1.4 a	17.5 ± 0.3 b	0.3 ± 0.0 c	4.8 ± 0.3 a	4.3 ± 0.1 a	0.3 ± 0.0 b	31.5 ± 0.9 a	24.5 ± 0.5 b		0.5 ± 0.0 c
Glu	40.1 ± 2.1 b	51.6 ± 1.7 a	5.4 ± 0.3 c	83.4 ± 5.3 b	97.4 ± 3.8 a	6.3 ± 0.1 c	49.3 ± 2.5 a	55.2 ± 2.3 a	23.5 ± 0.7 b	102.8 ± 4.2 a	103.5 ± 5.2 a		35.7 ± 0.7 b
Gly	11.1 ± 0.3 b	11.9 ± 0.3 a	0.7 ± 0.1 c	23.9 ± 1.8 a	15.6 ± 0.4 b	1.1 ± 0.0 c	2.5 ± 0.0 a	1.9 ± 0.1 b	4.3 ± 0.1 c	26.3 ± 2.8 a	20.2 ± 0.3 b		4.7 ± 0.1 c
Ala	13.6 ± 0.4 a	14.4 ± 0.2 a	1.4 ± 0.2 b	38.1 ± 3.6 a	19.3 ± 0.1 b	1.7 ± 0.1 c	3.0 ± 0.1 b	2.2 ± 0.0 c	5.8 ± 0.1 a	36.1 ± 3.0 a	30.5 ± 0.4 b		7.0 ± 0.3 c
Cys	1.4 ± 0.1 b	1.6 ± 0.0 a	0.1 ± 0.0 c	N.d.	0.6 ± 0.1 a	0.1 ± 0.0 b	0.2 ± 0.0 c	0.7 ± 0.0 a	0.3 ± 0.0 b	N.d.	N.d.		0.31 ± 0.0 a
Val	14.0 ± 0.7 a	14.7 ± 0.5 a	1.4 ± 0.2 b	31.1 ± 1.8 a	22.0 ± 0.8 b	1.7 ± 0.0 c	5.5 ± 0.2 b	4.7 ± 0.2 c	8.6 ± 0.3 a	37.3 ± 0.6 a	32.0 ± 1.0 b		8.4 ± 0.1 c
Met	0.9 ± 0.0 b	1.6 ± 0.1 a	N.d.	7.0 ± 0.4 a	0.9 ± 0.0 b	0.03 ± 0.0 c	N.d.	N.d.	N.d.	1.7 ± 0.0 a	1.6 ± 0.1 b		0.1 ± 0.0 c
Ile	9.0 ± 0.6 b	10.9 ± 0.4 a	0.8 ± 0.0 c	25.5 ± 1.6 a	17.0 ± 0.5 b	1.0 ± 0.0 c	6.1 ± 0.4 a	6.9 ± 0.3 a	6.5 ± 0.3 a	28.3 ± 0.4 a	24.2 ± 0.7 b		5.9 ± 0.0 c
Leu	19.9 ± 0.7 a	21.0 ± 0.7 a	1.5 ± 0.0 b	26.7 ± 1.0 b	33.0 ± 1.5 a	1.9 ± 0.0 c	10.1 ± 0.4 b	9.4 ± 0.4 b	12.7 ± 0.7 a	58.3 ± 1.2 a	48.5 ± 0.9 b		12.5 ± 0.2 c
Tyr	8.2 ± 0.4 a	8.6 ± 0.3 a	0.5 ± 0.1 b	17.0 ± 1.1 a	13.7 ± 0.6 b	0.6 ± 0.0 c	4.2 ± 0.2 a	4.5 ± 0.2 a	4.8 ± 0.6 a	24.4 ± 0.4 a	20.3 ± 0.8 b		4.6 ± 0.9 c
Phe	11.6 ± 0.5 b	15.3 ± 0.5 a	1.0 ± 0.0 c	24.9 ± 1.4 a	24.9 ± 0.8 a	1.2 ± 0.1 b	12.0 ± 0.4 b	15.4 ± 0.6 a	9.3 ± 0.0 c	39.0 ± 0.3 a	33.3 ± 1.0 b		9.3 ± 0.2 c
Lys	9.1 ± 0.2 a	9.3 ± 0.4 a	0.4 ± 0.01 b	34.5 ± 1.5 a	11.4 ± 0.4 b	0.6 ± 0.1 c	2.6 ± 0.0 a	1.4 ± 0.5 bc	1.8 ± 0.2 ab	23.9 ± 0.4 a	14.7 ± 0.1 b		1.9 ± 0.1 c
His	5.6 ± 0.1 b	5.9 ± 0.0 a	0.3 ± 0.0 c	11.3 ± 0.9 a	9.9 ± 0.6 a	0.3 ± 0.0 b	2.2 ± 0.1 a	2.0 ± 0.0 a	1.5 ± 0.1 b	16.8 ± 0.1 a	13.5 ± 0.9 b		1.6 ± 0.2 c
Arg	14.4 ± 0.9 a	14.4 ± 0.5 a	0.3 ± 0.0 b	32.4 ± 2.3 a	19.9 ± 0.5 b	0.3 ± 0.0 c	4.2 ± 0.3 a	3.8 ± 0.2 a	0.7 ± 0.1 b	36.1 ± 0.9 a	27.8 ± 0.6 b		0.5 ± 0.0 c
Pro	12.0 ± 0.5 b	16.1 ± 0.5 a	1.8 ± 0.0 c	51.3 ± 1.3 a	31.3 ± 1.3 b	2.2 ± 0.0 c	19.1 ± 0.6 b	23.3 ± 1.0 a	9.3 ± 0.2 c	55.4 ± 0.5 a	51.0 ± 1.8 b		12.1 ± 0.3 c
Total	211.5 ± 9.3 b	237.9 ± 6.1 a	17.2 ± 0.6 c	506.0 ± 21.2 a	373.3 ± 11.7 b	22.6 ± 1.8 c	130.6 ± 5.1 a	139.6 ± 5.9 a	94.6 ± 2.4 b	590.5 ± 7.7 a	499.3 ± 5.1 b		112.1 ± 1.8 c

N.d. = Not detected. The different letters marked in the table indicate a significant difference between the groups of data (*p* < 0.05).

## Data Availability

Data are contained within the article.

## Supplementary Materials

The following supporting information can be downloaded at: https://www.mdpi.com/article/10.3390/antiox14050538/s1, Figure S1. Circular dichroism Spectra of (A) albumins, (B) globulins, (C) prolamins, and (D) glutelins in raw, roasted, and burnt malt.
