# Supplementary material for: Improvement of the Structure and Antioxidant Activity of Protein–Polyphenol Complexes in Barley Malts Using Roasting Methods
Source: Antioxidants (Basel). 2025 Apr 29;14(5):538. doi: 10.3390/antiox14050538 (PMC12108492; doi:10.3390/antiox14050538)
Supplement: Supplementary file 1 [file antioxidants-14-00538-s001.zip › Supplementary material Figure S1.pdf]

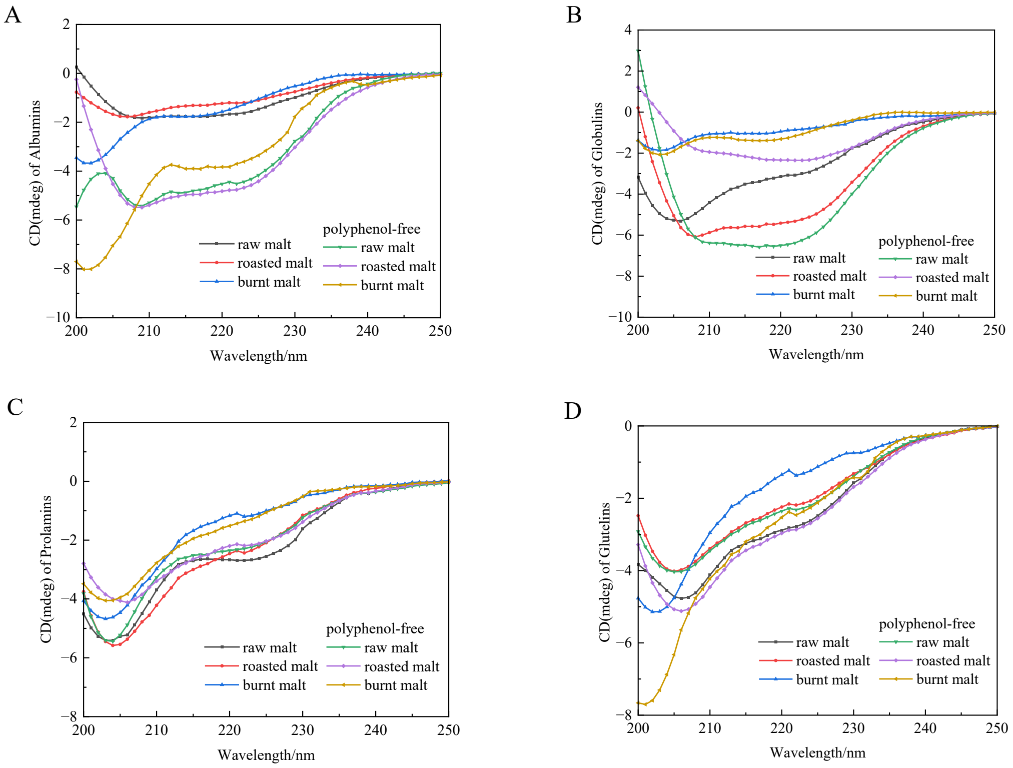

Figure S1 Circular dichroism Spectra of (A) albumins, (B) globulins, (C) prolamins, and (D) glutelins in raw, roasted, and burnt malt.
