# Supplementary figures and images for: Improvement of the Structure and Antioxidant Activity of Protein–Polyphenol Complexes in Barley Malts Using Roasting Methods
Source: Antioxidants (Basel). 2025 Apr 29;14(5):538. doi: 10.3390/antiox14050538 (PMC12108492; doi:10.3390/antiox14050538)

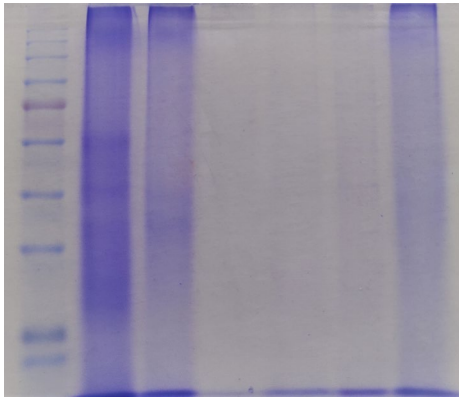

I

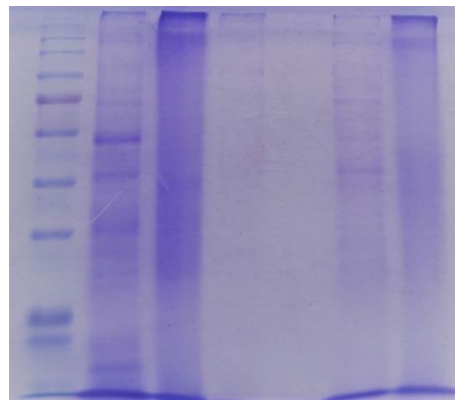

II

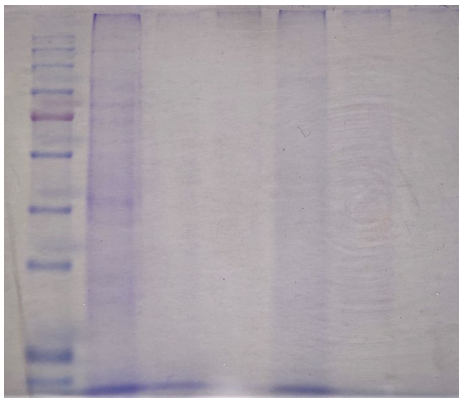

III

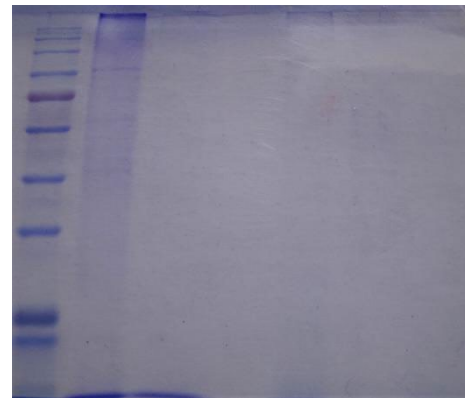

IV

The original SDS-PAGE pictures.

Supplement: Supplementary file 1 [file antioxidants-14-00538-s001.zip › Supplementary material SDS-PAGE.pdf]
